# Two Distinct Cardiolipin Synthases Operate in *Agrobacterium tumefaciens*

**DOI:** 10.1371/journal.pone.0160373

**Published:** 2016-07-29

**Authors:** Simon Czolkoss, Christiane Fritz, Georg Hölzl, Meriyem Aktas

**Affiliations:** 1 Microbial Biology, Ruhr University Bochum, Bochum, Germany; 2 Institute of Molecular Physiology and Biotechnology of Plants (IMBIO), University of Bonn, Germany; Academia Sinica, TAIWAN

## Abstract

Cardiolipin (CL) is a universal component of energy generating membranes. In most bacteria, it is synthesized via the condensation of two molecules phosphatidylglycerol (PG) by phospholipase D-type cardiolipin synthases (PLD-type Cls). In the plant pathogen and natural genetic engineer *Agrobacterium tumefaciens* CL comprises up to 15% of all phospholipids in late stationary growth phase. *A*. *tumefaciens* harbors two genes, *atu1630* (*cls1*) and *atu2486* (*cls2*), coding for PLD-type Cls. Heterologous expression of either *cls1* or *cls2* in *Escherichia coli* resulted in accumulation of CL supporting involvement of their products in CL synthesis. Expression of *cls1* and *cls2* in *A*. *tumefaciens* is constitutive and irrespective of the growth phase. Membrane lipid profiling of *A*. *tumefaciens* mutants suggested that Cls2 is required for CL synthesis at early exponential growth whereas both Cls equally contribute to CL production at later growth stages. Contrary to many bacteria, which suffer from CL depletion, *A*. *tumefaciens* tolerates large changes in CL content since the CL-deficient *cls1*/*cls2* double mutant showed no apparent defects in growth, stress tolerance, motility, biofilm formation, UV-stress and tumor formation on plants.

## Introduction

Bacterial cytoplasmic membranes consist of various lipids with distinct chemical and physical properties. They are arranged in a two-dimensional matrix with proteins embedded, thus allowing selective transport, sensing, communication and energy-generating processes. The most abundant prokaryotic membrane lipids are the glycerophospholipids phosphatidylethanolamine (PE), phosphatidylglycerol (PG) and cardiolipin (CL). CL is an anionic phospholipid with varying amounts in bacterial membranes. Despite its low abundance during exponential growth, CL influences diverse physiological processes, such as localization and stability of proteins and protein complexes, formation of membrane microdomains and the generation of membrane potential [1–5]. The molecular structure of CL is unique. Due to its four acyl chains and a small hydrophilic headgroup, CL displays a cone-shaped architecture and preferably locates at regions of negative membrane curvature [6–8].

In many bacteria, CL helps to withstand environmental stresses. *Escherichia coli* accumulates CL when cells are exposed to high salinity and CL mediates localization and activity of the osmosensory transporter ProP [2]. Temperature-dependent CL synthesis has been documented for *Bacillus subtilis* under hypoxic conditions [9]. In *Staphylococcus aureus*, prolonged survival under high-salinity conditions depends on the presence of CL [10]. In *Rhodobacter sphaeroides*, CL deficiency leads to altered cell morphology and negatively affects biofilm formation [11]. However, despite its broad impact on cell physiology, CL does not appear to be essential for bacterial survival, since loss of CL mostly results in rather mild phenotypes.

Many bacteria harbor multiple genes coding for possible CL synthases (Cls) suggesting genetic redundancy [12]. *E*. *coli* harbors three Cls paralogues encoded by *clsA*, *ybhO* (*clsB*) and *ymdC* (*clsC*) [13]. In *B*. *subtilis*, three homologues to *E*. *coli clsA* have been identified, termed *ywnE* (*clsA*), *ywjE*, and *ywiE* [7]. CL synthesis in *S*. *aureus* is mediated by the gene products of *cls1* and *cls2* [14].

Traditionally, CL biosynthesis pathways were classified as either eukaryotic or prokaryotic. The most prevalent eukaryotic pathway is driven by CDP-alcohol phosphatidyltransferases (CAP-type Cls) and involves the condensation of PG with cytidine diphosphate-diacylglycerol (CDP-DAG) to form CL. In contrast, the transphosphatidylation between two PG molecules via Cls enzymes of the phospholipase D superfamily (PLD-type Cls) has been considered as a hallmark of prokaryotic CL synthesis. However, recent findings suggest that the classification into eukaryotic and prokaryotic CL biosynthesis pathways is not appropriate anymore as enzymes with characteristics of eukaryotic Cls have been identified in some prokaryotes and vice versa. Phylogenetic analysis revealed a large number of unicellular eukaryotes containing putative PLD-type Cls [12]. At the same time, bacterial Cls utilizing PG and another molecule for CL biosynthesis rather than two PG molecules have been discovered. Sco1389 from *Streptomyces coelicolor* was the first bacterial Cls identified to use CDP-DAG as a phosphatidyl donor in CL synthesis [15]. The bifunctional PLD-type Cls (Xc_0186) of *Xanthomonas campestris* is able to use CDP-DAG and PG for CL production and CDP-DAG and ethanolamine for PE synthesis [16]. *E*. *coli* ClsC catalyzes CL formation by a transesterification reaction between PE and PG, thus representing yet another pathway for CL biosynthesis [13].

In the phytopathogenic soil bacterium *Agrobacterium tumefaciens* CL accumulates to up to 15% of the total phospholipids in late stationary phase. Apart from the common bacterial phospholipids PE, PG and CL, *A*. *tumefaciens* contains the methylated PE derivatives monomethyl-PE, dimethyl-PE and PC. Additionally, it produces two phosphorus-free ornithine lipids (OL1/2) and under phosphate deprivation different glycolipids and diacylglycerol trimethylhomoserine (DGTS) [17, 18]. *A*. *tumefaciens* is widely known for its ability to cause crown gall tumors upon infection of plant tissues. Tumorigenesis is based on the transfer of a small part of bacterial DNA, the T-DNA, via the type IV secretion system (T4SS) into the plant cell [19]. Tumor formation strongly correlates with the membrane phospholipid composition. PC is required for host-microbe interaction whereas the absence of ornithine lipids stimulates tumor formation [18, 20]. These findings raised the question of whether CL plays a role in *Agrobacterium* physiology and virulence. Therefore, we set out to identify CL biosynthesis enzymes and to examine their physiological importance in *A*. *tumefaciens*.

## Materials and Methods

### Bacterial strains, plasmids and growth conditions

The strains, plasmids and oligonucleotides used in this study are listed in S1 Table and S2 Table. If not stated otherwise, *Escherichia coli*, *Agrobacterium tumefaciens* C58 and derivative *cls* mutant strains (Δ*cls1*, Δ*cls2* and Δ*cls1*/Δ*cls2*) were grown in LB medium [21] supplemented with kanamycin (50 μg/ml), ampicillin (100 μg/ml) or tetracycline (10 μg/ml) if appropriate. *E*. *coli* DH5α was used for cloning procedures. *E*. *coli* BL21 (DE3) served as a host for the overproduction of Cls1 and Cls2 from the corresponding pET24b-based expression plasmids. Growth temperature was 37°C for *E*. *coli* strains and 30°C for *A*. *tumefaciens* strains if not stated otherwise. For the induction of the virulence cascade, *A*. *tumefaciens* was grown to an OD_600_ of 0.2 in AB medium (pH 5.5, supplemented with 1% (w/v) glucose) [22] prior to the addition of acetosyringone (Sigma-Aldrich, St. Louis, MO, USA) to a final concentration of 0.1 mM. Cells were further incubated for 18 h at 23°C.

### Plasmid construction

Recombinant DNA work was carried out according to standard protocols [21]. For the heterologous expression of *A*. *tumefaciens cls1* and *cls2* in *E*. *coli* both genes were PCR amplified using chromosomal DNA as a template and the designated primers S2 Table). The PCR products were digested with the engineered restriction sites NdeI and XhoI and cloned into the expression vector pET24b, resulting in pBO3712 and pBO3713. For promoter activity analysis, PCR-generated fragments of the expected promoter regions of *cls1* (280 bp) and *cls2* (465 bp) were digested with KpnI and XhoI and ligated into pAC01 treated with the same enzymes resulting in pBO1256 and pBO3732. Chromosomal deletions of *cls1* and *cls2* were created as previously described using the suicide vector pK19*mobsacB* [18]. For the construction of the respective plasmids, 400–500 bp fragments up- and downstream of *cls1* and *cls2* were amplified using the designated primer pairs (S2 Table). PCR products of the upstream regions were digested with EcoRI and PstI and cloned into the vector pK19*mobsacB*, resulting in the plasmids pBO1254 (pK19*mobsacB*_cls1_up) and pBO1255 (pK19*mobsacB*_cls2_up). PCR-generated fragments of *cls1* and *cls2* downstream regions were digested with PstI and HindIII and ligated into pBO1254 and pBO1255, respectively, resulting in pBO1270 (pK19*mobsacB*_cls1_up_down) and pBO1271 (pK19*mobsacB*_cls2_up_down). For the complementation of the *cls* deletion strains, *cls1* (1577 bp) and *cls2* (1481 bp) coding regions were cloned into the pBBSyn vector, using the restriction sites XbaI and SacI, resulting in pBO3723 and pBO3724. Expression of *cls1* and *cls2* was under the control of the strong constitutive *P*_*syn*_ promoter. The correct nucleotide sequences of all generated plasmids were confirmed by automated sequencing. Plasmids were transferred into *E*. *coli* via transformation using heat shock and into *A*. *tumefaciens* via electroporation.

### Construction of *cls* deletion mutants

Chromosomal deletions of *cls1* and *cls2* were engineered using the plasmids pBO1270 and pBO1271, which cannot replicate in *A*. *tumefaciens*. Both constructs were transferred into *A*. *tumefaciens* via electroporation. Kanamycin-resistant clones should have integrated the plasmids by a first recombination event and were cultivated for 18 h in LB medium. Different dilutions (10^−2^, 10^−3^ and 10^−4^) were plated on LB agar plates containing 10% (w/v) sucrose. Double cross over events resulted in sucrose tolerant and kanamycin sensitive clones. Double deletion of *cls* genes was achieved using the *cls1* single deletion strain as a background for mutagenesis of *cls2* in an analogous manner. Sucrose-resistant and kanamycin-sensitive clones were analyzed using PCR and Southern blot analysis [21].

### Overproduction of Cls1 and Cls2 in *E*. *coli*

For the overexpression of *cls1* and *cls2* in *E*. *coli*, pBO3712 and pBO3713 were transferred into *E*. *coli* BL21(DE3) and plated on LB agar containing kanamycin. After incubation for 18 h at 37°C, single colonies were transferred into fresh LB medium with kanamycin and incubated overnight. Cells were again transferred into fresh medium containing kanamycin to a start OD_580_ of 0.1. Protein synthesis was induced with 0.4 mM IPTG (isopropyl-β-d-thiogalactopyranoside) at an OD_580_ of 0.6 for 3 h at 30°C. Equal amounts of cells were harvested and protein and lipid samples were further processed.

### *In vitro* activity of Cls1 and Cls2 in *Agrobacterium* crude extracts

*A*. *tumefaciens* wild type and *cls* deletion strains were grown in LB medium for 18 h at 30°C. 30 ml of cells were harvested, washed and resuspended in 3 ml Tris/HCl buffer (50 mM Tris, 50 mM NaCl, pH8). Cell lysis was achieved by French Press (three passes, 900 psi). 100 μl of reaction mixture contained 300 μg of total protein, 0.4 mM of the respective potential lipid substrate(s) (PE, PG, CDP-DAG, CL,18:1) and 0.02% Triton X-100. Samples were incubated for 12 hours at 30°C before lipids were extracted according to Bligh and Dyer [23] and analyzed via one-dimensional thin layer chromatography (1D-TLC) as described below.

### Lipid analysis by TLC

Phospholipids of *A*. *tumefaciens* or *E*. *coli* strains were isolated according to Bligh and Dyer [23]. Briefly, 1 to 2 ml of culture were harvested by centrifugation, washed with 500 μl of water to remove residues of medium and resuspended in 100 μl of water. 375 μl of a mixture of methanol:chloroform (2:1) was added and samples were homogenized before 100 μl of water and 100 μl of chloroform were added. Samples were briefly vortexed and centrifuged for 5 minutes at 13.000 rpm. The lower organic phase was collected and dried under vacuum. The lipid pellet was resuspended in 15 μl of methanol:chloroform (1:1) and spotted onto a HPTLC silica gel 60 plate (Merck, Darmstadt, Germany). For 1D-TLC a mixture of *n*-propanol:propionate:chloroform:water (3:2:2:1) was used as a running solvent. For two-dimensional TLC (2D-TLC), mixtures of chloroform:methanol:water (65:25:4) and chloroform:methanol:acetic acid:water (90:15:10:3,5) were used as running solvents for first and second dimension, respectively. For the visualization of the lipids, plates were sprayed with molybdenum blue reagent (Sigma-Aldrich, St. Louis, MO, USA) or charred after CuSO_4_-treatment at 180°C. Purified phospholipid standards (bovine heart cardiolipin, 1,2-dioleoyl-sn-glycero-3-phospho-rac-(1-glycerol), L-α-phosphatidylethanolamine) were purchased from Merck (Darmstadt, Germany).

### Lipid analysis by gas chromatography MS

Lipids were isolated, separated and analyzed as previously described [17, 24]. Briefly, cells were grown in rich medium to early stationary phase before lipids were isolated and separated using 2D-TLC as stated above. Gas chromatography (GC) MS was used to quantify their fatty acid methyl esters.

### Western blotting

For protein analysis, 1 ml of *A*. *tumefaciens* or *E*. *coli* cells was harvested and resuspended in SDS loading buffer in relation to the final OD_580_ (*E*. *coli*) or OD_600_ (*A*. *tumefaciens*). An OD value of 1 thereby equaled 100 μl SDS loading buffer. Samples were heated to 95°C for 10 min before equal amounts of extracts were separated by sodium dodecyl sulfate (SDS)-12,5%-polyacrylamide gel electrophoresis (SDS-PAGE) followed by Western blot analysis using anti-Penta-His antibody (Qiagen, Hilden, Germany) and chemiluminescence detection (ECL). Detection of VirB8 and VirB9 was achieved using *A*. *tumefaciens* specific antisera (VirB8: 1/100.000; VirB9: 1/10.000).

### Potato disc infection assay

Quantitative tumorigenesis assays with potato tuber discs were carried out as described before [25]. Briefly, *A*. *tumefaciens* was grown to an OD_600_ 0.9–1.0 in rich medium, harvested via centrifugation and resuspended in phosphate-buffered saline at 10^8^ and 10^6^ cells/ml for inoculation. Potatoes were peeled and disinfected by immersing in 0.625% sodium hypochlorite before discs of approximately 0.4 cm thickness were cut from cylinders. The discs were placed on water agar and infected with 10 μl of bacterial suspensions (10^8^ and 10^6^ cells/ml). Petri dishes were sealed and incubated at 23°C for two days. The discs were then placed on water agar supplemented with 100 μg/ml ticarcillin to kill bacteria and incubated at 23°C for three weeks before tumors were counted.

### *β*-Galactosidase assays

The *β*-galactosidase activity of *A*. *tumefaciens* cells transformed with pBO1256 and pBO3732 grown with ampicillin and tetracycline in LB medium or LB medium with additional 0.4 M NaCl was quantified according to standard protocols [26]. The plasmid pAC01 containing the promoterless *lacZ* gene was used as the negative control.

## Results and Discussion

### *Agrobacterium tumefaciens* encodes two putative cardiolipin synthases

**Fig 1:**
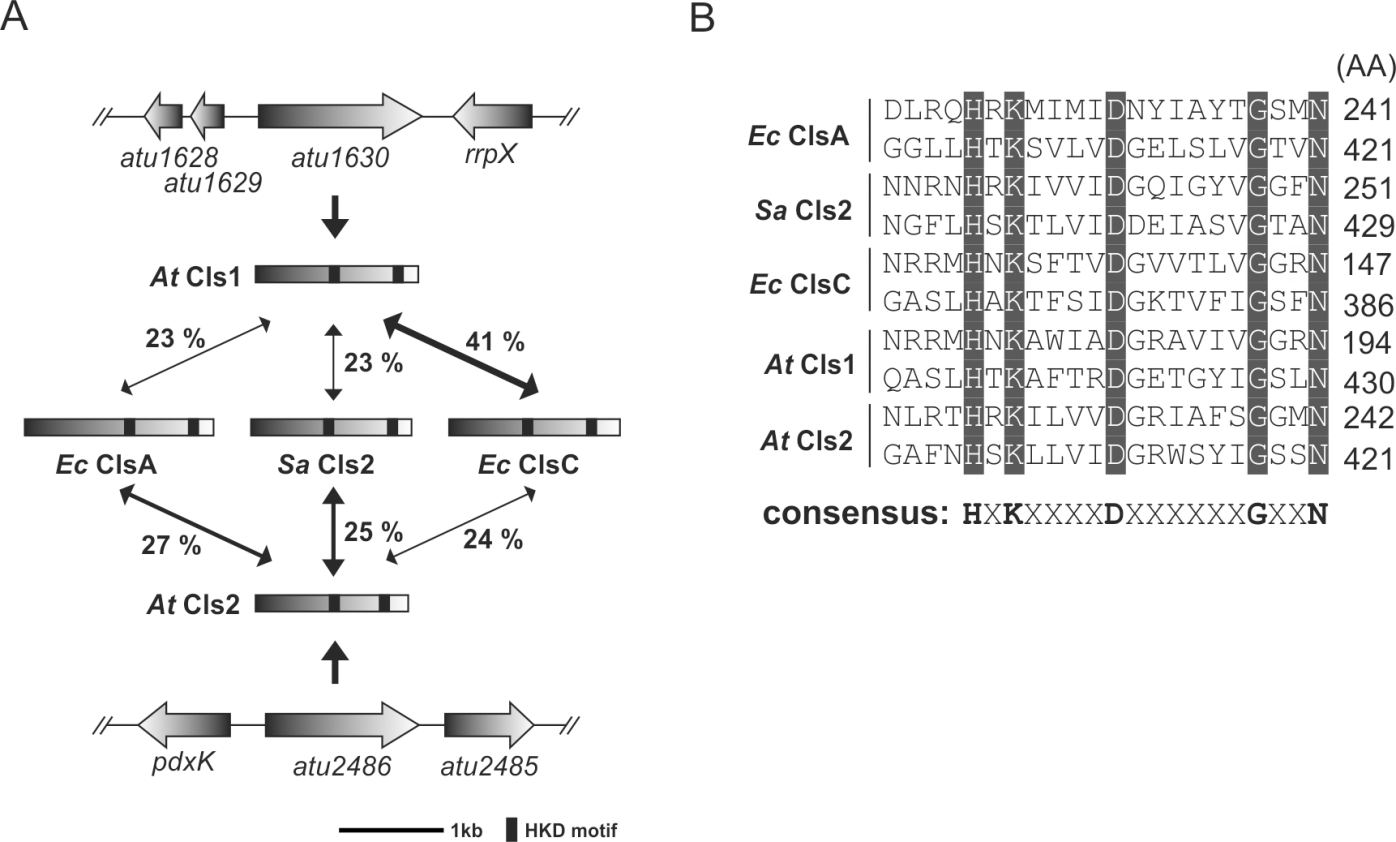
Identification of putative cardiolipin synthases in *Agrobacterium tumefaciens*. **(A)** Sequence identity of *A*. *tumefaciens* Cls1 and Cls2 with characterized Cls from *E*. *coli* and *S*. *aureus*. The *A*. *tumefaciens cls* genes *atu1630* (*cls1*) and *atu2486* (*cls2*) are located on the circular chromosome. **(B)** Alignment of HKD-motifs of different Cls enzymes. Conserved amino acids are highlighted in gray and the consensus HKD-motif is indicated. Abbreviations: *rrpx* (two component response regulator); *pdxK* (pyridoxamine kinase); *Ec* (*E*. *coli*); *Sa* (*S*. *aureus*)*; At* (*A*. *tumefaciens*). GeneBank accession number: *atu1630* (NP_354623.1); *atu2486* (NP_355434.2); Ec *clsA* (NP_415765.1); Ec *clsC* (NP_415564.2); Sa *cls2* (WP_000571560.1).

Using the protein sequences of well characterized cardiolipin synthases (Cls) of the PLD- and CAP-type as query, we identified two open reading frames (*atu1630* and *atu2486*) on the circular chromosome of *A*. *tumefaciens*, encoding PLD-type Cls homologs. On the basis of our functional analysis (see below) and in the order of their chromosomal location, we renamed *atu1630* to *cls1* and *atu2486* to *cls2*. The *cls1* gene codes for a polypeptide with a predicted molecular mass of approximately 58 kDa. With about 54 kDa, the translated open reading frame of *cls2* is slightly shorter. The deduced Cls1 and Cls2 proteins display only about 23% sequence identity (data not shown) whereas Cls1 is about 41% identical to ClsC from *E*. *coli* (Fig 1A). Cls2 displays moderate sequence identities to the housekeeping Cls of *E*. *coli* (ClsA; ~ 27%) and *S*. *aureus* (Cls2; ~ 25%) as predicted by Clustal 2.1 (Percent Identity Matrix) [27]. The *S*. *aureus* and *E*. *coli* enzymes most likely use two PG molecules for CL synthesis and represent generic PLD-type cardiolipin synthases [14, 28]. *A*. *tumefaciens* Cls1 and Cls2 contain two HKD-motifs characteristic for enzymes of the phospholipase D (PLD) superfamily (Fig 1B). This group contains both eukaryotic and prokaryotic enzymes of phospholipid synthesis and modification [29, 30]. The highly conserved amino acids of the HKD-motifs [HXK(X)_4_D(X)_6_G(X)_2_N] are crucial for enzyme activity [13, 16, 31] and are thought to form a single active site [32]. Additionally, *A*. *tumefaciens* Cls1 harbors one N-terminal transmembrane domain (TMD, residues 1 to 24) whereas Cls2 features two TMDs (residues 4 to 26 and residues 39 to 61), as predicted by TMHMM software [33, 34]. According to these predictions, the active sites of *Agrobacterium* Cls1 and Cls2 face the periplasmic side of the membrane. This also holds true for the *B*. *subtilis* and *R*. *sphaeroides* Cls (data not shown) and has been proposed for *S*. *aureus* and *E*. *coli* Cls enzymes [14, 35]. Thus, a periplasmic active side might be a common feature of PLD-type Cls. Like *A*. *tumefaciens* Cls1 and Cls2, many bacterial Cls contain two (*S*. *aureus* Cls2, *B*. *subtilis* ClsA and *E*. *coli* ClsA) or three (*B*. *subtilis* YwiE) putative TMDs. However, the membrane associated *E*. *coli* ClsB and ClsC proteins lack TMDs and might represent peripheral Cls [13].

### *Agrobacterium cls1* and *cls2* encode functional cardiolipin synthases

**Fig 2:**
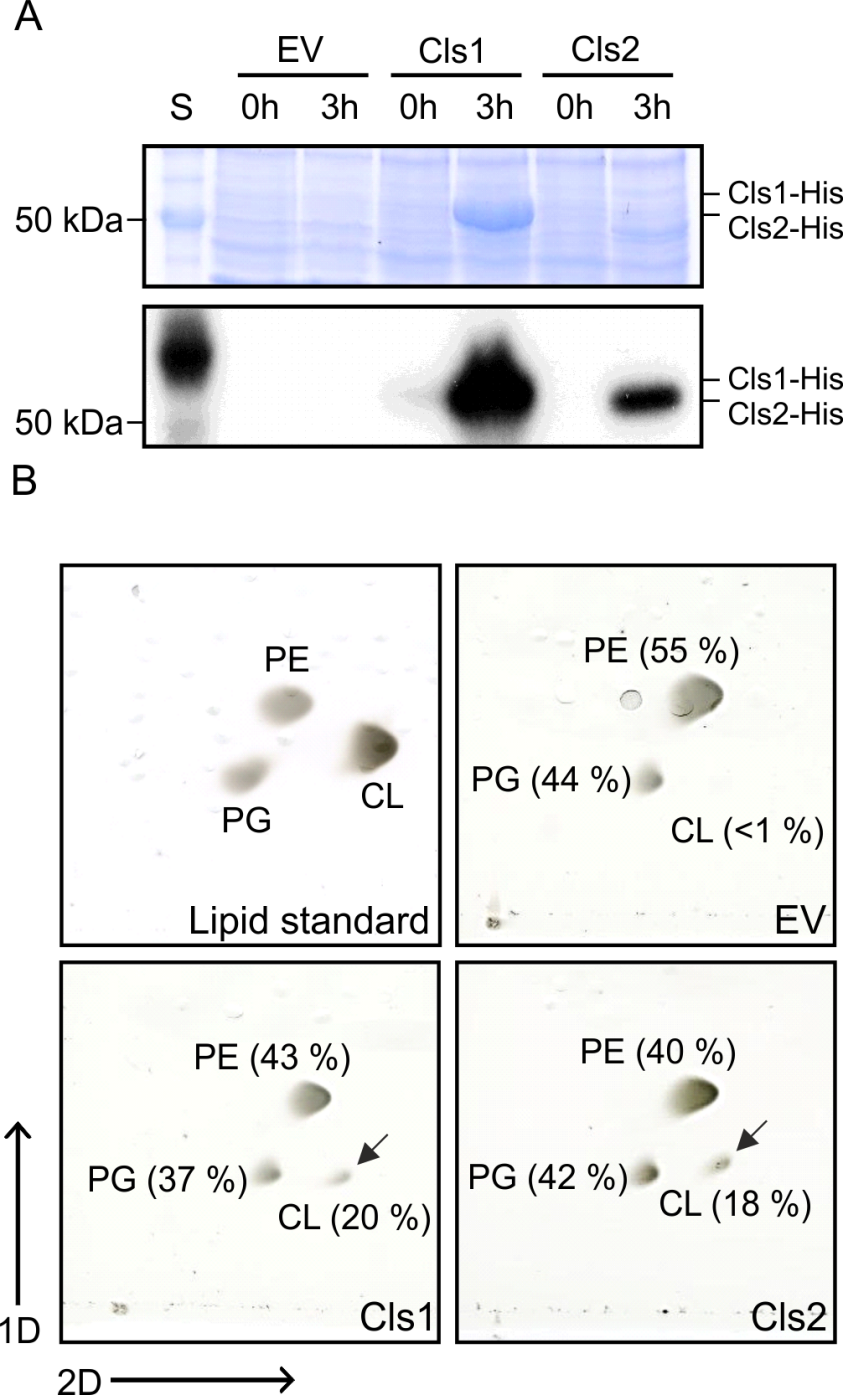
Phospholipid profile of *E*. *coli* strains expressing *A*. *tumefaciens cls1* and *cls2*. **(A)** SDS-PAGE (upper panel) and Western blot (lower panel) of cell extracts from *E*. *coli* BL21(DE3) strains producing Cls1-His and Cls2-His. *E*. *coli* strains were grown in LB medium and expression of *cls1* and *cls2* was induced with 0.4 mM IPTG for 3h at 30°C. A strain carrying empty vector pET24b was used as a control (EV). Total proteins were separated by SDS-PAGE, and Cls1-His and Cls2-His were detected by Western analysis using a His-tag specific antibody. **(B)** Phospholipid analysis of Cls1 and Cls2 producing *E*. *coli* strains. Total lipids of *E*. *coli* strains expressing either *cls1* or *cls2* were isolated and separated by two-dimensional thin-layer chromatography (2D-TLC). Accumulation of CL is marked by arrows and absence of CL is indicated by a dashed circle. Commercial PE, PG and CL were used as lipid standards. Lipids were visualized by CuSO_4_-treatement. Relative intensities of phospholipid spots were determined using Alpha Ease FC software. Abbreviations: S (BenchMark protein ladder); PE (phosphatidylethanolamine); PG (phosphatidylglycerol); CL (cardiolipin).

To provide evidence that *A*. *tumefaciens cls1* and *cls2* encode active Cls, both genes were heterologously expressed in *E*. *coli* BL21(DE3) as C-terminally His-tagged variants. Synthesis of both proteins with calculated molecular masses of approximately 59 kDa (Cls1) and 55 kDa (Cls2) was confirmed by Western blotting analysis (Fig 2A).

During logarithmic growth *E*. *coli* produces marginal amounts of CL as a result of low expression of *clsA*, whose product is responsible for the most part of CL production [13, 36]. Thus, we analyzed Cls1 and Cls2 activity in *E*. *coli* during logarithmic growth by two-dimensional thin-layer chromatography (2D-TLC). In contrast to the strain containing the empty vector (EV), strains expressing *cls1* or *cls2* produced notable amounts of CL (Fig 2B, lower panel) providing evidence that *cls1* and *cls2* encode functional Cls.

Attempts to purify Cls1 and Cls2 failed due to formation of inclusion bodies, which impeded *in vitro* biochemical characterization of the enzymes. Removal of the N-terminal putative TMD of Cls1 did not improve protein solubility (data not shown). However, the truncated Cls1 variant was still active (S1 Fig), demonstrating that the putative membrane-spanning region is not crucial for Cls1 activity. Likewise, TMDs of the *Enterococcus faecium* S44 and of the *E*. *coli* Cls enzymes are dispensable for enzyme activity and membrane association [37–39]. It has been proposed that the N-terminal TMDs in PLD-type Cls enzymes are cleaved off during maturation [38, 39], but their precise role remains unknown.

### An *Agrobacterium tumefaciens cls1*/*cls2* double mutant is deficient in CL formation

**Fig 3:**
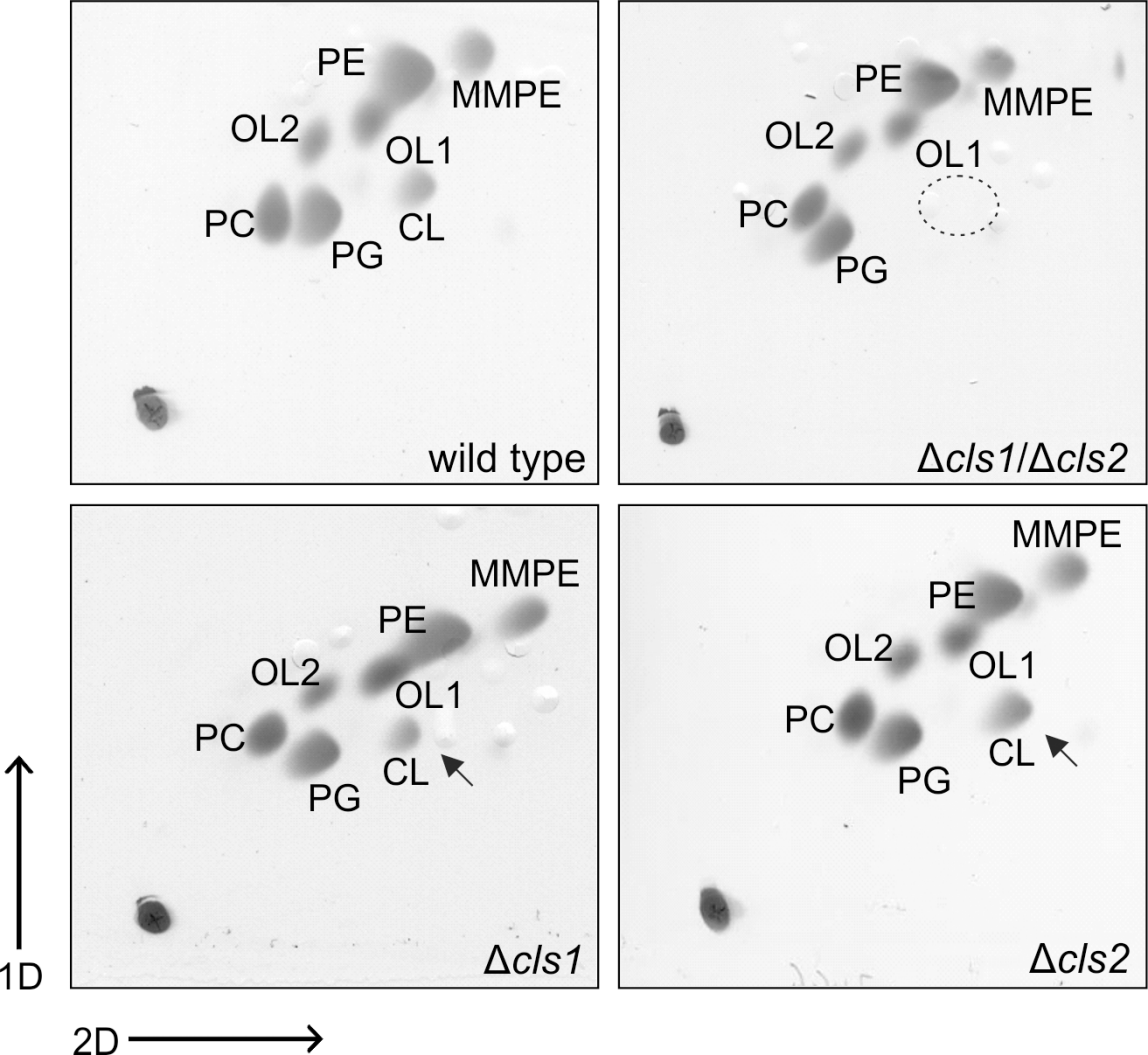
Lipid profiles of *Agrobacterium tumefaciens* wild type and *cls* mutant strains. Cells were cultivated in LB medium grown until late exponential phase (OD ~ 4) Total lipids of *A*. *tumefaciens* wild type, Δ*cls1*, Δ*cls2* and Δ*cls1*/Δ*cls2* were isolated and analyzed by 2D-TLC. Loss of CL in the Δ*cls1*/Δ*cls2* strain is indicated by a dashed circle and accumulation of CL is marked by arrows. Lipids were visualized by CuSO_4_-treatement. Abbreviations: PE (phosphatidylethanolamine); MMPE (monomethyl-PE); OL1 (ornithine lipid 1); OL2 (ornithine lipid 2); PC (phosphatidylcholine); PG (phosphatidylglycerol); CL (cardiolipin).

To investigate the physiological role and contribution of Cls1 and Cls2 to CL synthesis in *A*. *tumefaciens*, we constructed isogenic *cls1* and *cls2* single deletion strains (Δ*cls1* and Δ*cls2*) and a double mutant (Δ*cls1*/Δ*cls2*). The lipid profiles of stationary phase wild type and *cls* mutant strains were investigated by 2D-TLC (Fig 3). As previously shown, *A*. *tumefaciens* wild type contained the phospholipids PE, PG and CL, the methylated PE-derivatives monomethyl-PE (MMPE) and PC as well as the ornithine lipids OL1 and OL2 (Fig 3, upper left panel) [17, 18]. The lipid profiles of the single *cls1* and *cls2* mutants resembled that of the wild type, suggesting that Cls1 and Cls2 act redundantly in CL synthesis (Fig 3, lower panel). The double mutant lacked detectable amounts of CL (Fig 3, upper right panel) suggesting that Cls1 and Cls2 are responsible for CL formation in *A*. *tumefaciens*. Expression of plasmid-encoded *cls1* or *cls2* restored CL accumulation to wild-type-like levels (S2 Fig). As mentioned above, *A*. *tumefaciens* Cls1 shares the highest sequence identity with the *E*. *coli* ClsC (41%; Fig 1A) which utilizes PE and PG for CL formation. Interestingly, ClsC activity is dependent on its neighboring gene *ymdB* which is unique among Cls [13]. The role of YmdB (a regulator of RNase III cleavage) [40] in CL formation still remains obscure. The *ymdB-clsC* operon seems to be conserved in some *E*. *coli*-related bacteria [13]. However, *A*. *tumefaciens* lacks a YmdB homolog and Cls1 does not seem to require an additional factor for its activity.

**Fig 4:**
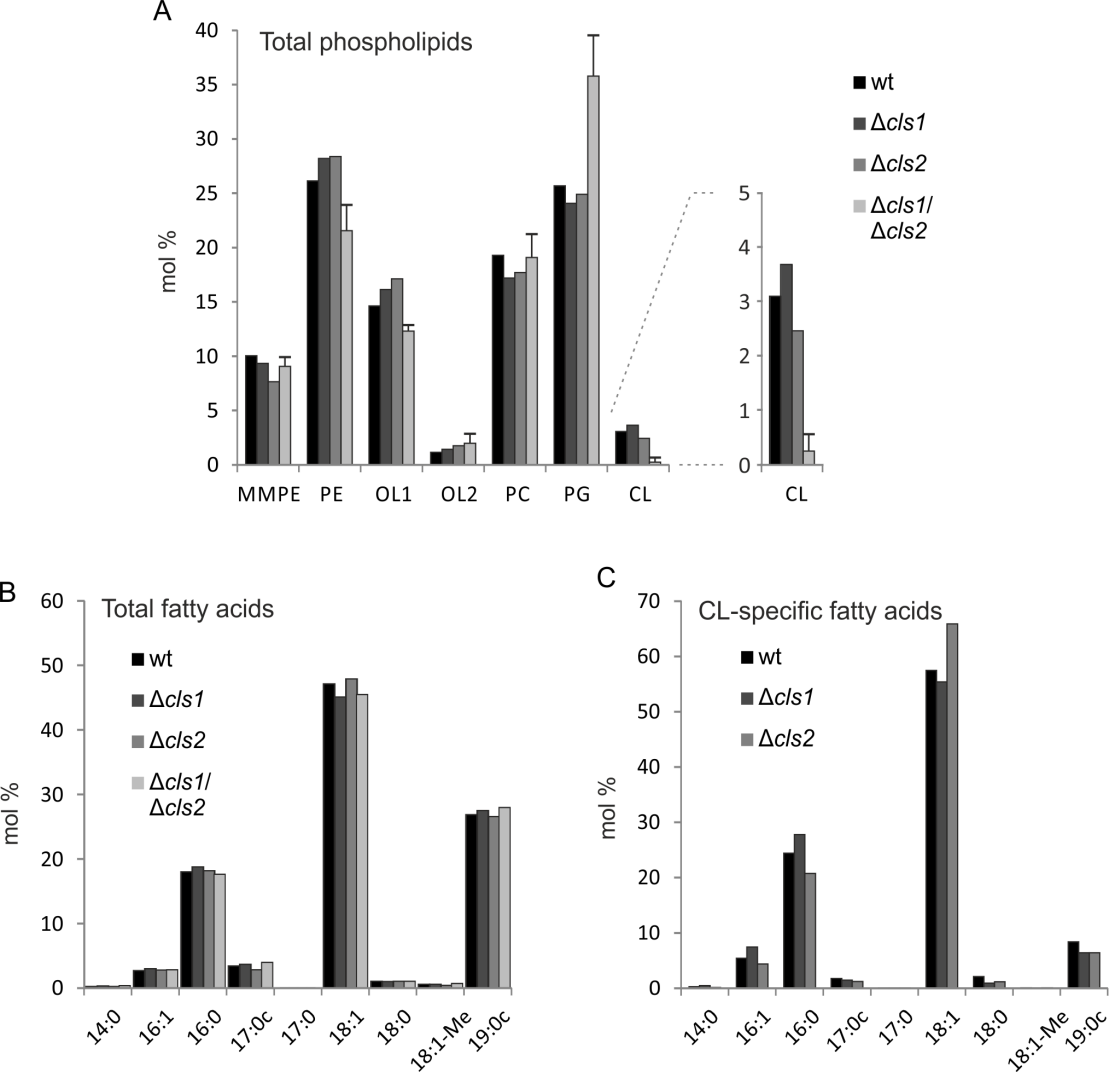
Gas chromatography MS analysis of membrane lipids from different *Agrobacterium tumefaciens* strains. Cultures were harvested in early exponential phase before lipids were isolated, separated and analyzed as previously described [17]. Mass spectrometry (MS) was used to quantify the lipid **(A)** and total fatty acid compositions **(B)** of *A*. *tumefaciens* wild type and *cls* single and double mutants via fatty acid methyl esters. **(C)** Fatty acid composition of cardiolipins in different *A*. *tumefaciens* strains. CL was separated from other lipids by 2D-TLC. MS was used to identify CL-specific fatty acids. Data represent single measurements except for Δ*cls1*/Δ*cls2* in (A) with n = 2. Abbreviations: wt (wild type); PE (phosphatidylethanolamine); MMPE (monomethyl-PE); OL1 (ornithine lipid 1); OL2 (ornithine lipid 2); PC (phosphatidylcholine); PG (phosphatidylglycerol); CL (cardiolipin).

For a more detailed analysis of the membrane composition of the different *A*. *tumefaciens cls* mutant strains, we conducted quantitative GC/MS analysis (Fig 4). Lipid species were characterized by the quantification of fatty acid methyl esters as previously described [17, 24]. Overall lipid profiles from early stationary phase *cls* single mutants were similar to the wild type, confirming the results obtained by TLC analysis (Fig 4A). The quantities of the different phospholipids and OLs were consistent with previous reports [20, 41]. In the wild type, PE and PG make up to ~ 50% of total lipids. While PC accounts for ~ 20%, its precursor lipid molecule MMPE is found in smaller amounts (around 10%). The phosphorus-free lipid OL1 accounts for around ~ 15% of total lipid while its hydroxylated derivative OL2 occurs only in traces. The CL content reaches up to ~ 5% in *A*. *tumefaciens* wild type under the tested conditions (Fig 4A). The double mutant displayed slightly decreased PE and OL1 content and barely detectable amounts of CL (~0.2%) compared to the wild type and the single mutants. The residual CL in the double mutant might be explained by additional not yet identified Cls or promiscuity of other phospholipid synthases. Similarly, minor CL amounts (~0,5%) are still detected in *R*. *sphaeroides cls* mutant [42].The PG levels were substantially increased in the *A*. *tumefaciens cls1/cls2* mutant (Fig 4A) supporting its role as precursor for CL formation by Cls1 and Cls2. Similar shift in phospholipid content have been documented in *cls* mutants of other organisms, such as *Saccharomyces cerevisiae*, *S*. *aureus* and *E*. *coli* [10, 13, 43].

We further investigated whether the loss of tetraacylic CL affects the fatty acid profile of *A*. *tumefaciens*. As illustrated in Fig 4B, the membrane lipid fatty acid composition of all strains was comparable to the wild type and contained primarily saturated or mono-unsaturated acyl chains. 18:1 was the most common fatty acid in all strains followed by 19:0 cyclopropane (19:0c) and 16:0 fatty acids. Altogether, they accounted for more than 90% of total fatty acids. Additionally, small amounts of mono-unsaturated 16:1 and 17:0 cyclopropane (17:0c) could be detected (< 5%). 18:0 and 18:1-Me were only present in traces. These numbers are well in line with previous reports [17] and imply that the composition of fatty acids in *A*. *tumefaciens* membrane lipids is not influenced by the loss of CL. This is in contrast, to the situation in *Streptococcus mutans* where loss of CL results in elevated levels of saturated fatty acids at the expense of unsaturated fatty acids [44].

Assuming that Cls1 and Cls2 might produce CL species with different fatty acid composition, we took a closer look at the CL-specific fatty acids in the wild type and the *cls* single mutants (Fig 4C). In the wild type, about 60% of fatty acids were mono-enoic fatty acids with 18 carbons followed by ~ 25% of unsaturated 16:0. 16:1 and 19:0c accounted for less than 10%. A similar CL fatty acid distribution was found in the individual *cls1* and *cls2* mutants suggesting that both enzymes produce similar CL products by using the same pool of substrates. An important aspect of bacterial CL synthesis is that it is a reversible reaction depending on substrate availability and product requirement [35, 45]. Thus, PLD-type Cls might also be involved in CL degradation and turnover. It is therefore possible, that while both *A*. *tumefaciens* Cls1 and Cls2 make use of the same pool of substrates, they might differ in their role in CL formation and turnover.

### Contribution of Cls1 and Cls2 to CL production is growth phase-dependent

**Fig 5:**
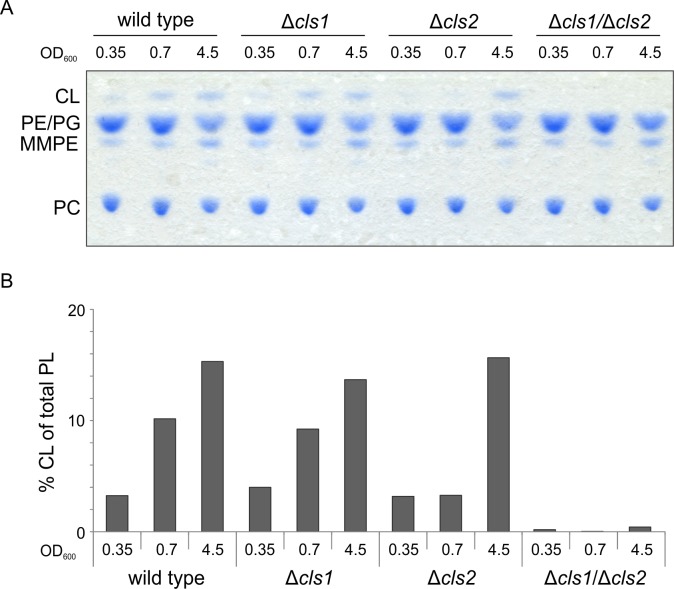
Growth phase dependent accumulation of CL. *A*. *tumefaciens* wild type and *cls* mutant strains were grown in LB medium and samples for lipid analysis were taken at the indicated optical densities. **(A)** Lipid composition was examined using 1D-TLC. Lipids were visualized using molybdenum-blue spray. **(B)** Quantification of CL content. Intensities of CL spots were determined using Alpha Ease FC software. Experiments were done in triplicate and one representative result is shown. Abbreviations: PE (phosphatidylethanolamine); MMPE (monomethyl-PE); PC (phosphatidylcholine); PG (phosphatidylglycerol); CL (cardiolipin).

The amount of CL in the membranes can significantly vary during the cell cycle as a consequence of increased or decreased Cls activity or accelerated CL turnover [14, 46–48]. We therefore compared CL formation of *A*. *tumefaciens* wild type and the *cls* mutant strains at different optical densities via 1D-TLC (Fig 5A). The relative CL content increased in a growth-phase dependent manner in the wild type and the Δ*cls* single deletion strains (Fig 5B). The double mutant lacked detectable CL regardless of the growth phase (Fig 5A), confirming that Cls1 and Cls*2* are primarily responsible for CL formation in *A*. *tumefaciens*. It is striking that CL levels in the Δ*cls2* strain did not increase until growth exceeded the logarithmic growth phase, suggesting that Cls1 cannot compensate for the absence of Cls2 at early stationary phase. This distinct contribution of Cls1 and Cls2 to CL synthesis dependent on growth phase suggests differences in their role and regulation and might be attributed to different transcriptional or enzymatic activities.

### The c*ls1* and *cls2* genes are constitutively expressed

**Fig 6:**
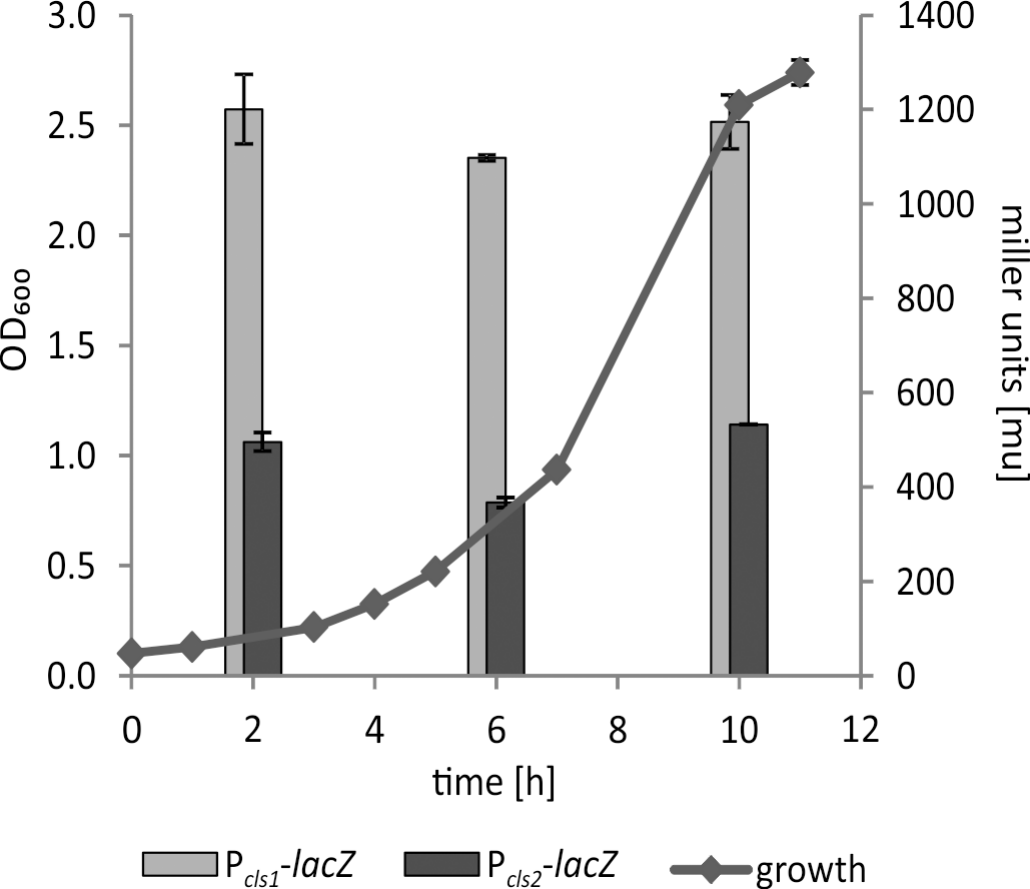
Expression of *cls1*- and *cls2-lacZ* fusions in *A*. *tumefaciens*. *A*. *tumefaciens* wild-type strains carrying pBO1256 (P_*cls1*_-*lacZ*) and pBO3732 (P_*cls2*_-*lacZ*) were cultivated in LB medium. *β*-Galactosidase activities were determined after 2, 6 and 10 hours of incubation. Results represent means and SD of three independent experiments.

To test whether the growth phase-dependent accumulation of CL mediated by Cls1 and Cls2 is reflected by differential expression, we constructed translational *cls1-* and *cls2*-*lacZ* fusions. In the wild type, expression of both *cls-lacZ* fusions was constitutive and completely independent of the growth phase (Fig 6). Overall expression of *cls1* was higher than expression of *cls2* under all tested conditions. Deletion of either *cls1* or *cls2* did not affect expression of the remaining *cls* (data not shown). The constitutive expression of both *cls* genes suggests that growth phase-dependent control of CL biosynthesis occurs at the post-transcriptional level. Likewise, accumulation of CL in the stationary phase in *E*. *coli* is attributed to changes in ClsA activity whereas *clsA* expression remains at a constant level throughout growth. Additionally, the stationary phase increase in ClsA activity was irrespective of the phospholipid environment, as is the case in strains lacking *pssA1* (encoding for a temperature-sensitive phosphatidylserine synthase) and *pgsA3* (encoding for phosphatidylglycerol-phosphate synthase) [47]. Different contribution of two Cls enzymes in CL homeostasis has also been reported in *S*. *aureus*. Here, CL accumulation in stationary phase and after phagocytosis is caused by the activity of Cls2 rather than Cls1 and might be caused by different regulation of *cls1* and *cls2* mRNA synthesis and/or turnover [14].

In many bacteria, CL accumulates in response to osmotic stress [2, 46]. Thus, we checked whether *cls1* and *cls2* expression or CL accumulation is affected by high salt concentrations in *A*. *tumefaciens*. Neither expression of *cls1* and *cls2* (data not shown) nor CL production were influenced under osmotic stress conditions (S3 Fig). Thus, CL accumulation is not regulated by osmotic stress in *A*. *tumefaciens*.

### Cls2 utilizes PG for CL formation *in vitro*

**Fig 7:**
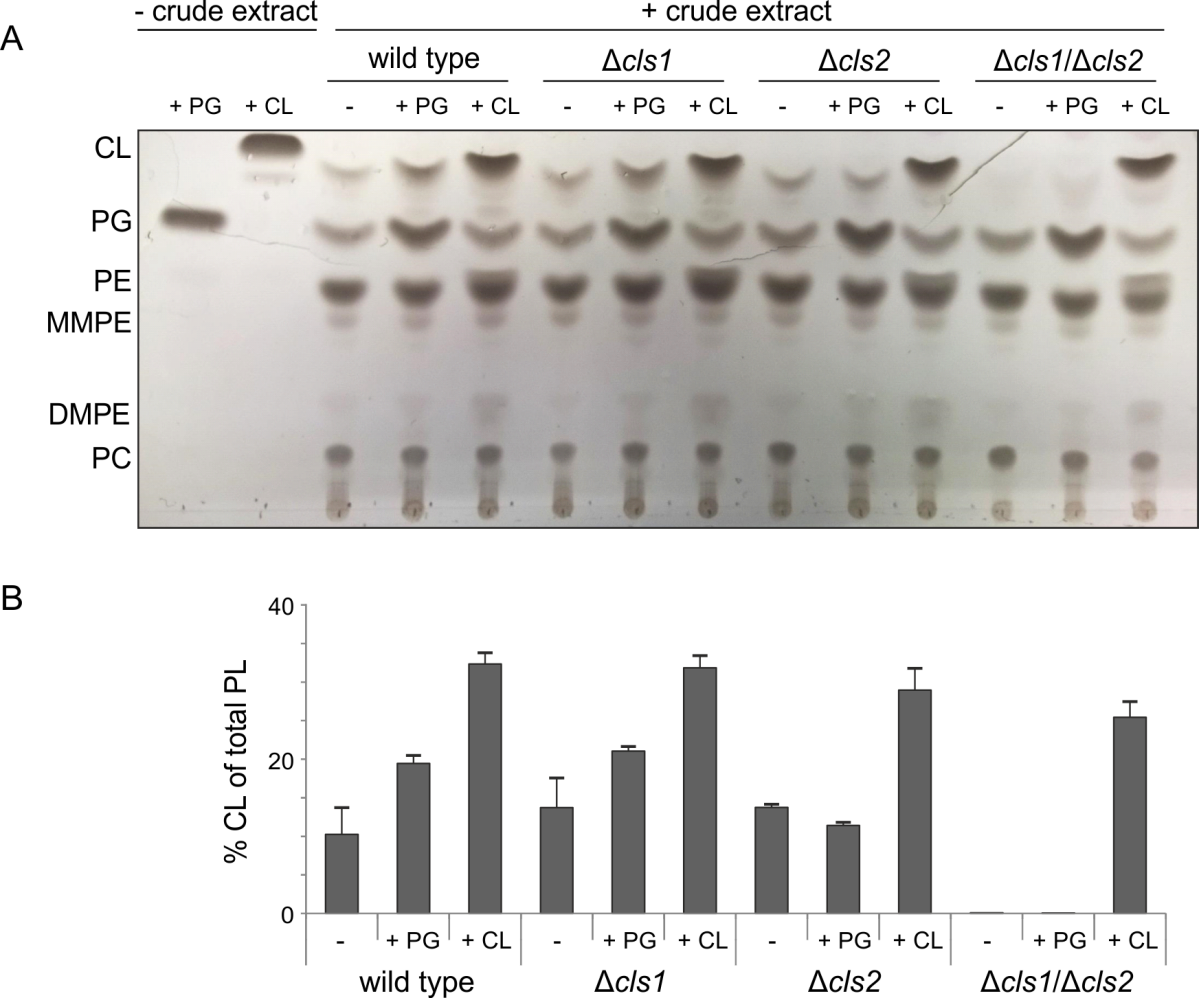
*In vitro* activity of Cls1 and Cls2 in crude extracts. Crude extracts of *A*. *tumefaciens* wild type and *cls* mutant strains were used for Cls *in vitro* assays. 100 μl of cell lysates with 300 μg total protein were incubated with potential lipid substrates (0.4 mM) in micellar form before total lipids were isolated and examined by 1D-TLC **(A)**. Lipids were visualized by CuSO_4_-treatment. **(B)** Relative intensities of CL spots were determined using Alpha Ease FC software. Experiments were done at least three times and one representative result is shown. Abbreviations: PE (phosphatidylethanolamine); MMPE (monomethyl-PE); DMPE (dimethyl-PE); PC (phosphatidylcholine); PG (phosphatidylglycerol); CL (cardiolipin).

Since Cls1/2 purification from *E*. *coli f*ailed, we aimed to characterize the activity and substrate specificity of the Cls1 and Cls2 enzymes by assaying their activities in crude extracts of *A*. *tumefaciens* wild type and *cls* deletion strains. Cell lysates of the strains were supplemented with commercially available potential lipid substrates (PG, PE or CDP-DAG) alone or in combination, and reaction products were isolated and analyzed via 1D-TLC. PE or CDP-DAG were not converted to CL under all tested conditions (data not shown). Remarkably, the CL content increased only when reaction mixture with wild type or Δ*cls1* cell lysates were supplemented with PG (Fig 7A and 7B). PG-dependent increase of CL was not detected in Δ*cls2* or Δ*cls1*/Δ*cls2* lysates suggesting that PG-dependent CL accumulation is mediated by Cls2. Although accumulation of PG in the double *cls* mutant but not in the single mutants (Fig 4A) indicated that both enzymes might utilize PG as substrate, a conversion of external PG to CL by Cls1 was not detectable (Fig 7). Differences in PG-species selectivity or requirement of specific cofactor(s) might account for these differences. It has been proposed that CL formation by PLD-type Cls is a reversible reaction. Degradation of CL by PLD-type Cls enzymes is dependent on substrate availability and product requirement [45]. Under our tested conditions, there was no evidence for turnover of the supplied CL.

### CL is not required for basic cellular processes and tumorigenesis in *A*. *tumefaciens*

**Fig 8:**
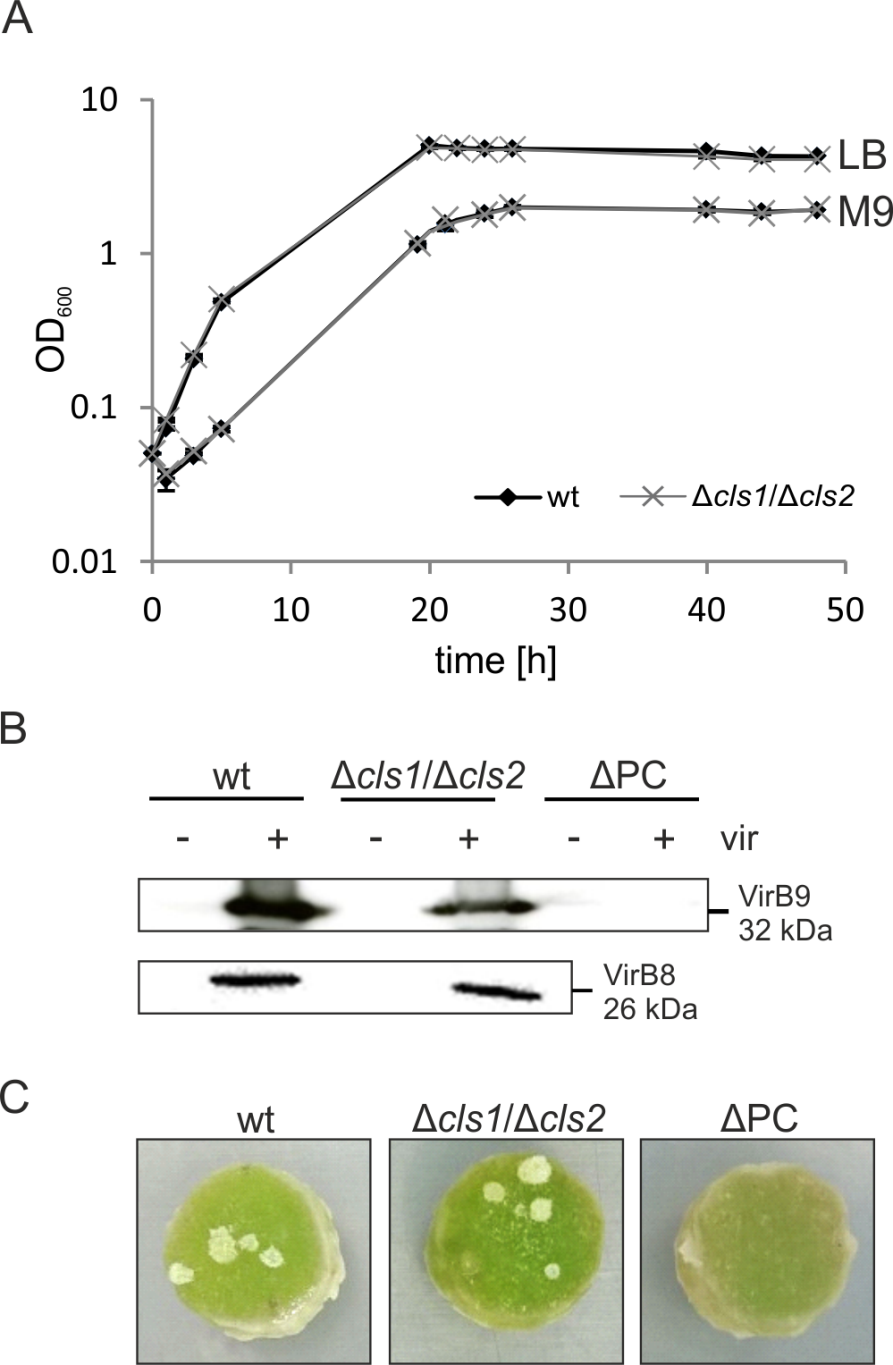
Phenotypic characterization of *A*. *tumefaciens* wild type and Δ*cls1*/Δ*cls2*. **(A)** Growth of *A*. *tumefaciens* wild type and a *cls1*/*cls2* double mutant. Strains were cultivated in LB or M9 minimal medium and cell density was monitored over two days. **(B)** Detection of the type IV secretion system components VirB8 and VirB9 in different *A*. *tumefaciens* strains. Cells were grown under non-virulence-inducing conditions (- vir) or virulence-inducing conditions (+ vir) in AB minimal medium. Total proteins were separated via SDS-PAGE and screened for the presence of VirB8 and VirB9 using Western blot analysis with specific antisera. A strain lacking PC (ΔPC) was used as a negative control. **(C)** Representative images of *A*. *tumefaciens*-induced tumorigenesis on potato discs. Discs were infected with 10^8^ cells and incubated for 21 days before images were taken.

Anionic phospholipids, like CL and PG, are crucial for cell cycle, osmotic stress and cell morphology in different bacteria [3, 10, 11, 46]. To determine the physiological role of CL in *A*. *tumefaciens*, we conducted experiments addressing the viability of the CL-deficient mutant strain. Loss of CL did not affect growth in rich or minimal medium (Fig 8A). Neither the absence of CL, nor the deletion of either *cls1* or *cls2* impaired biofilm formation or motility (data not shown). Notably, growth of the Δ*cls1*/Δ*cls2* strain under UV-stress, at elevated or decreased temperatures and in the absence or presence of up to 0.4 M sodium chloride was not compromised (data not shown) suggesting that CL is not required for survival of *A*. *tumefaciens* under those conditions. We have previously shown that *A*. *tumefaciens* lacking PC exhibits severe defects in tumorigenesis, which is due to the absence of the membrane-spanning type IV secretion system (T4SS) [18, 49]. To investigate whether CL is also involved in pathogenicity, we monitored the presence of VirB8 and VirB9, which are components of the T4SS (Fig 8B). In the wild type and the CL-deficient mutant, both VirB8 and VirB9 were detected after treatment with the artificial virulence inductor acetosyringone (+ vir), demonstrating that CL, in contrast to PC, is not necessary for the synthesis of the T4SS. Tumorigenesis studies on potato discs further revealed that the CL-deficient mutant is not impaired in tumor formation (Fig 8C). In contrast, contribution of CL to virulence has been demonstrated in a number of human pathogens such as *Moraxella catarrhalis*, *S*. *aureus* and *Salmonella enterica* [10, 31, 50, 51].

A recent study revealed that CL deficiency in *R*. *sphaeroides* leads to defects in biofilm formation and produces ellipsoidal-shaped cells, while growth remained unaffected [11]. Likewise, a *Pseudomonas putida cls* mutant is smaller in size compared to wild-type cells and exhibits increased sensitivity to several antibiotics but displays no growth defect [52]. However, cell shape or size of the CL-deficient strain was unaffected (data not shown) suggesting a negligible role of CL in cell morphology in *A*. *tumefaciens*.

Loss of CL in *A*. *tumefaciens* is accompanied by increased PG levels. Like CL, PG is an anionic phospholipid contributing to membrane potential and stability in different organisms [53]. CL deficiency in *E*. *coli* is accompanied by increased PG levels at the cell poles compensating the anionic and polar function of CL in the membrane [8]. Thus, it is tempting to believe that elevated amounts of PG compensate for the lacking CL in the Δ*cls1*/Δ*cls2* strain.

The present study was intended to understand metabolic pathways and physiological relevance of CL in *A*. *tumefaciens*. We showed that *A*. *tumefaciens* harbors two distinctly regulated PLD-type enzymes responsible for CL formation. Loss of CL was not accompanied by obvious phenotypic changes. In contrast to many other bacteria, *A*. *tumefaciens* seems to be more flexible in accommodating CL loss. Contrary to these bacteria, *Agrobacterium* membranes are characterized by a complex lipid composition including additional lipids such as MMPE, DMPE, PC and two ornithine lipids, which may compensate the loss of CL. The hydroxyl bound fatty acids in both ornithine lipids in *A*. *tumefaciens* contain almost exclusively a 19:0 cyclopropanted fatty acid [17, 20]. Similar to CL, membrane lipids containing cyclopropanated fatty acids are proposed to increase membrane fluidity [54, 55]. Thus, it is conceivable that the OLs in *A*. *tumefaciens* might compensate the function of CL in maintaining membrane fluidity. Furthermore, Vences-Guzman et al. showed that the relative amount of the head-group hydroxylated OL2 increases at lower growth temperature supporting a role of OL2 in membrane fluidity [20].

## Supporting Information

S1 Fig The N-terminal truncated Cls1 variant is still active. Total lipids from *E*. *co*li strains producing wild type Cls1 (Cls1 wt) or the N-terminal truncated version lacking the first 20 amino acids (ΔN20) were isolated after 4 h and 18 h of induction with 0.4 mM IPTG and analyzed by one-dimensional thin-layer chromatography (1D-TLC).
(TIF)

S2 Fig Complementation analysis of *A*. *tumefaciens* Δ*cls1*/Δ*cls2*. *A*. *tumefaciens* Δ*cls1*/Δ*cls2* was complemented with plasmid-encoded *cls1* (pBO3723) and *cls2* (pBO3724). Cells were harvested at the stationary phase and lipids were isolated and separated using 1D-TLC. Phospholipids were visualized using molybdenum blue staining. PE: phosphatidylethanolamine; MMPE: monomethyl-PE; PC: phosphatidylcholine; PG: phosphatidylglycerol; CL: cardiolipin.
(TIF)

S3 Fig Lipid profile of *A*. *tumefaciens* wild type in the presence of low and high NaCl concentrations. Cells were cultivated in LB medium or in LB medium with additional 0.4 M NaCl and harvested at early stationary phase. Total lipids were analyzed using 2D-TLC. Lipids were visualized by heating CuSO_4_-treated plates to 180°C. PE: phosphatidylethanolamine; MMPE: monomethyl-PE; OL1: ornithine lipid 1; OL2; ornithine lipid 2; PC: phosphatidylcholine; PG: phosphatidylglycerol; CL: cardiolipin.
(TIF)

S1 Table Strains and plasmids used in this study. (DOCX)

S2 Table Oligonucleotides used in this study. (DOCX)
