# Supplementary figures and images for: Two Distinct Cardiolipin Synthases Operate in Agrobacterium tumefaciens
Source: PLoS One. 2016 Jul 29;11(7):e0160373. doi: 10.1371/journal.pone.0160373 (PMC4966929; doi:10.1371/journal.pone.0160373)

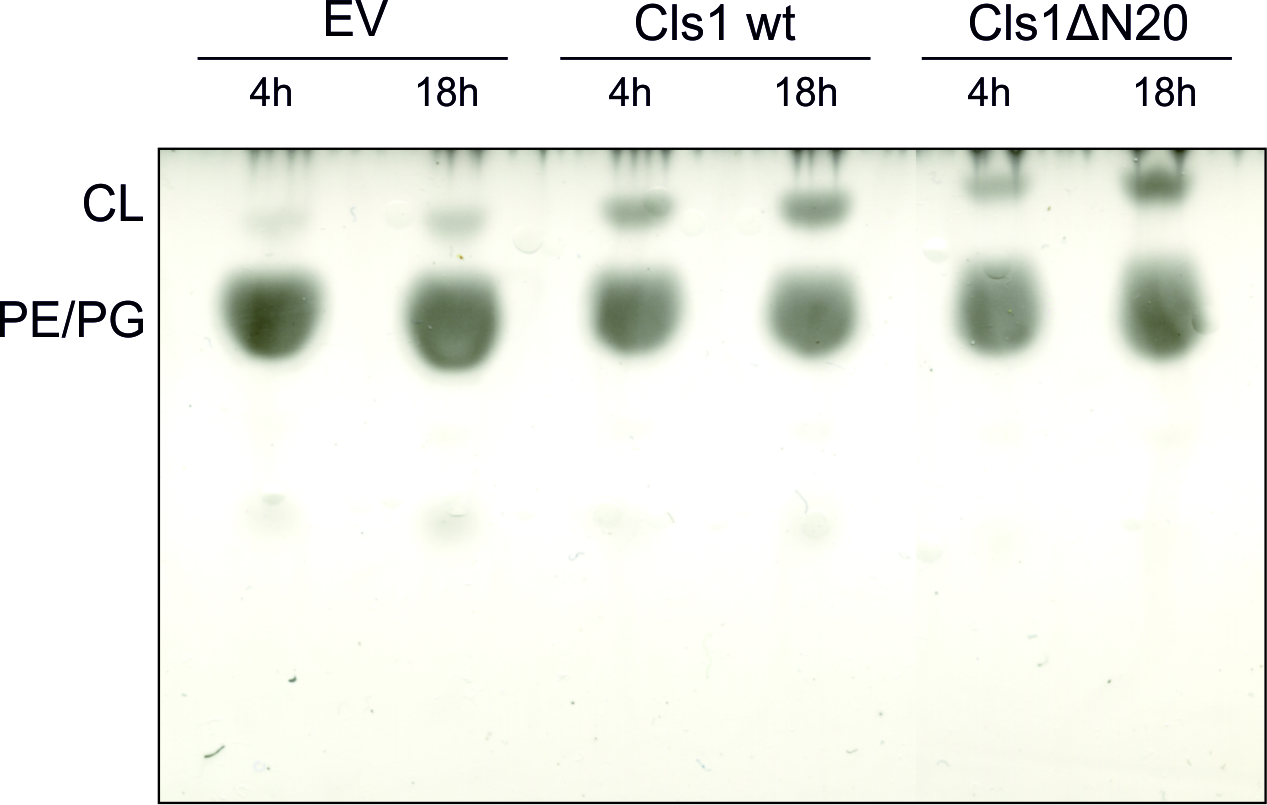

Supplement: S1 Fig — Total lipids from E. coli strains producing wild type Cls1 (Cls1 wt) or the N-terminal truncated version lacking the first 20 amino acids (ΔN20) were isolated after 4 h and 18 h of induction with 0.4 mM IPTG and analyzed by one-dimensional thin-layer chromatography (1D-TLC). (TIF) [file pone.0160373.s001.tif]

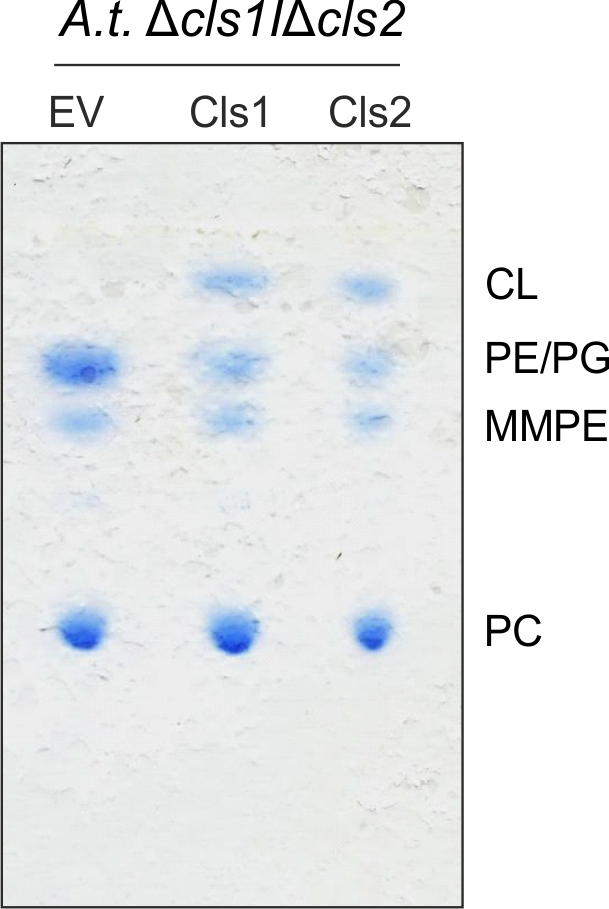

Supplement: S2 Fig — A. tumefaciens Δcls1/Δcls2 was complemented with plasmid-encoded cls1 (pBO3723) and cls2 (pBO3724). Cells were harvested at the stationary phase and lipids were isolated and separated using 1D-TLC. Phospholipids were visualized using molybdenum blue staining. PE: phosphatidylethanolamine; MMPE: monomethyl-PE; PC: phosphatidylcholine; PG: phosphatidylglycerol; CL: cardiolipin. (TIF) [file pone.0160373.s002.tif]

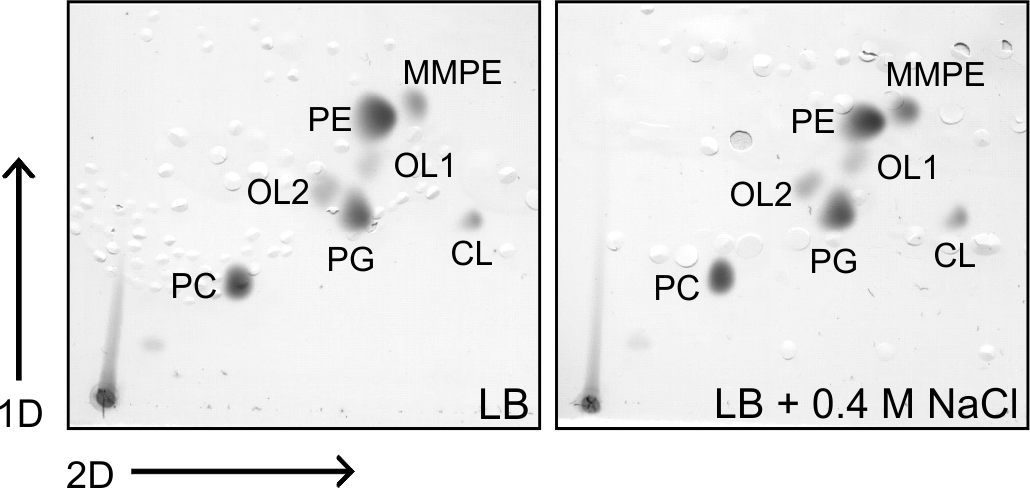

Supplement: S3 Fig — Cells were cultivated in LB medium or in LB medium with additional 0.4 M NaCl and harvested at early stationary phase. Total lipids were analyzed using 2D-TLC. Lipids were visualized by heating CuSO4-treated plates to 180°C. PE: phosphatidylethanolamine; MMPE: monomethyl-PE; OL1: ornithine lipid 1; OL2; ornithine lipid 2; PC: phosphatidylcholine; PG: phosphatidylglycerol; CL: cardiolipin. (TIF) [file pone.0160373.s003.tif]
