# Supplementary material for: Two Distinct Cardiolipin Synthases Operate in Agrobacterium tumefaciens
Source: PLoS One. 2016 Jul 29;11(7):e0160373. doi: 10.1371/journal.pone.0160373 (PMC4966929; doi:10.1371/journal.pone.0160373)
Supplement: S1 Table — (DOCX) [file pone.0160373.s004.docx]

**S1 Table. Bacterial strains and plasmids used in this study.**

| Strain or plasmid | | Relevant characteristics^a^ | Reference or source |
| --- | --- | --- | --- |
| **Strain** | |  |  |
| *Agrobacterium tumefaciens* C58 | Wild type | C. Baron, Montreal, Canada |  |
| *A. tumefaciens* Δ*cls1* | Wild type derivative, deletion of the *cls1* (*atu1630*) gene | This study |  |
| *A. tumefaciens* Δ*cls2* | Wild type derivative, deletion of the *cls2* (*atu2486*) gene | This study |  |
| *A. tumefaciens* Δ*cls1*/Δ*cls2* | Wild type derivative, deletion of the *cls1* and *cls2* genes | This study |  |
| *A. tumefaciens* Δ*pmtA*/Δ*pcs* | Wild type derivative, deletion of the *pmtA* and *pcs* genes | (Wessel *et al.*, 2006) |  |
| *Escherichia coli* DH5α | Cloning host | (Hanahan, 1983) |  |
| *E. coli* BL21(DE3) | Expression host | Novagen, Madinson, USA |  |
| **Plasmids** | |  |  |
| pET24b | Km^R^; vector for overproduction of His-tagged proteins | Novagen, Madison, USA |  |
| pBBSyn | Km^R^; vector used for complementation | (Giacomini *et al.*, 1994) |  |
| pK19*mobsacB* | Km^R^; suicide vector used for mutant construction | (Schäfer *et al.*, 1994) |  |
| pAC01 | Ap^R^ Tc^R^; IncP broad host range promoter-probe vector with lacZ reporter | (Liu *et al.*, 2008) |  |
| pBO1254 | Km^R^; derivative of pK19*mobsacB* carrying the upstream region of *cls1* | This study |  |
| pBO1255 | Km^R^; derivative of pK19*mobsacB* carrying the upstream region of *cls2* | This study |  |
| pBO1256 | Ap^R^ Tc^R^; derivative of pAC01 containing the promoter region of *cls1* | This study |  |
| pBO1270 | Km^R^; derivative of pK19*mobsacB* carrying the up- and downstream regions of *cls1* | This study |  |
| pBO1271 | Km^R^; derivative of pK19*mobsacB* carrying the up- and downstream regions of *cls2* | This study |  |
| pBO3712 | Km^R^; derivative of pET24b for overproduction of Cls1 with a C-terminal His-tag | This study |  |
| pBO3713 | Km^R^; derivative of pET24b for overproduction of Cls2 with a C-terminal His-tag | This study |  |
| pBO3715 | Km^R^; derivative of pET24b for overproduction of Cls1∆N20 with a C-terminal His-tag | This study |  |
| pBO3723 | Km^R^; derivative of pBBSyn carrying the *cls1* gene for complementation | This study |  |
| pBO3724 | Km^R^; derivative of pBBSyn carrying the *cls2* gene for complementation | This study |  |
| pBO3732 | Ap^R^ Tc^R^; derivative of pAC01 containing the promoter region of *cls2* | This study |  |

^a^Ap: ampicillin; Km: kanamycin; Tc: tetracycline
