# Supplementary material for: Two Distinct Cardiolipin Synthases Operate in Agrobacterium tumefaciens
Source: PLoS One. 2016 Jul 29;11(7):e0160373. doi: 10.1371/journal.pone.0160373 (PMC4966929; doi:10.1371/journal.pone.0160373)
Supplement: S2 Table — (DOCX) [file pone.0160373.s005.docx]

**S2 Table. Oligonucleotides used in this study.**

| Oligonucleotide | | | Sequence (5‘ - 3‘)^a^ | | |
| --- | --- | --- | --- | --- | --- |
| **Overexpression of *cls1* and *cls2*** | | |  | | |
| *Cls1-Nde-fw* | | AAAACATATGACTGCATTTACGGTCTTCATC | | |  |
| *Cls1-Nde-N20-fw* | | AAAACATATGTCGTCATCGGCAAACAGCGG | | |  |
| *Cls1-Xho-rv* | | AAAACTCGAGAAGCTGAGATTCAAGCGGCAG | | |  |
| *Cls2-Nde-fw* | | AAAACATATGCTCGATCTCGTCATTGCCTATTG | | |  |
| *Cls2-Xho-rv* | | AAAACTCGAGAAGATAGGGTGAAGCGAGCCAC | | |  |
| **LacZ-fusion construction** | | |  | | |
| *Cls1-fw-Kpni* | | | AAAAGGTACCCCCTTCACGCAGGG | | |
| *Cls1-rv-Xhoi* | | | AAAACTCGAGTGTTGTTTGTCGCTCC | | |
| *Cls2-fw-Kpni* | | | AAAAGGTACCCAGAACAGCCTTGACC | | |
| *Cls2-rv-Xhoi* | | | AAACTCGAGGATAAGACTATAGAGC | | |
| **Mutant construction** | | | |  | |
| *Cls1-up-fw-Ecori* | | AAAAGAATTCTCATTGATCCATATCCTTCAGC | | |  |
| *Cls1-up-rv-Psti* | | AAAACTGCAGTCACTGTTGTTTGTCGCTCC | | |  |
| *Cls1-dwn-fw-Psti* | | AAAACTGCAGATCATGCGTGTCC | | |  |
| *Cls1-dwn-rv-Hindiii* | | AAAAAAGCTTTTCCTCACCAATCACC | | |  |
| *Cls2-up-fw-Ecori* | | AAAAGAATTCCAGAACAGCCTTGACC | | |  |
| *Cls2-up-rv-Psti* | | AAAACTGCAGGCACTCAGCCGATC | | |  |
| *Cls2-dwn-fw-Psti* | | AAAACTGCAGGAAGTCATGGACG | | |  |
| *Cls2-dwn-rv-Hindiii* | | AAAAAAGCTTGCAAGATCGAGCAGG | | |  |
| **Mutant complementation** | |  | | |  |
| *Cls1-fw-Xbai* | | AAAATCTAGAGTGACTGCATTTACGGTCTTCATC | | |  |
| *Cls1-rv-Saci* | | AAAAGAGCTCTCAAAGCTGAGATTCAAGCGGC | | |  |
| *Cls2-fw-Xbai* | | AAAATCTAGAATGCTCGATCTCGTCATTGCCTATTG | | |  |
| *Cls2-rv-Saci* | | AAAAGAGCTCCTAAAGATAGGGTGAAGCGAGC | | |  |

**^a^**restriction sites are underlined
